# Supplementary material for: Cost effectiveness of empagliflozin in adult patients with chronic kidney disease in the Netherlands
Source: PLoS One. 2024 Dec 10;19(12):e0315509. doi: 10.1371/journal.pone.0315509 (PMC11630597; doi:10.1371/journal.pone.0315509)
Supplement: S1 Appendix — (DOCX) [file pone.0315509.s001.docx]

# **Supplementary Materials**

**Cost effectiveness of empagliflozin in adult patients with chronic kidney disease in the Netherlands**

Tanja Fens^1,2^¶ ([0000-0003-3995-447X](https://orcid.org/0000-0003-3995-447X)), Bart P.H. Slob^1,2^*¶ ([0009-0008-9125-0190](https://orcid.org/0009-0008-9125-0190)), Maaike Weersma^3^, Maarten J. Postma ([0000-0002-6306-3653](https://orcid.org/0000-0002-6306-3653))^1,2,4,5,6^, Cornelis Boersma ([0000-0002-1190-2638](https://orcid.org/0000-0002-1190-2638))^1,2,7^ and Lisa de Jong^1,2^ ([0000-0001-8814-0670](https://orcid.org/0000-0001-8814-0670))

1. Department of Health Sciences, University Medical Center Groningen, University of Groningen, The Netherlands
2. Health-Ecore Ltd, Groningen/ Zeist, The Netherlands
3. Boehringer Ingelheim bv, Amsterdam, The Netherlands
4. Department of Economics, Econometrics & Finance, Faculty of Economics & Business, University of Groningen, The Netherlands
5. Department of Pharmacology and Therapy, Faculty of Medicine, Universitas Airlangga, Indonesia
6. Center of Excellence in Higher Education for Pharmaceutical Care Innovation, Universitas Padjadjaran, Indonesia
7. Department of Management Sciences, Open University, Heerlen, The Netherlands

*Corresponding author:

E-mail: [bartslob@health-ecore.com](mailto:bartslob@health-ecore.com) (BS)

¶ These authors contributed equally to this work

# **Model structures of submodules**


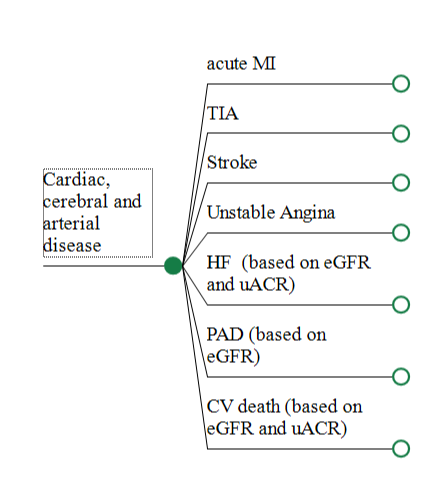


**Figure 1. Cardiovascular disease submodel.**

Abbreviations: CV, cardiovascular; eGFR, estimated glomerular filtration rate; HF, heart failure; MI, myocardial infarction; PAD, Peripheral arterial disease; TIA, transient ischaemic attack; uACR, urine albumin-creatinine ratio.


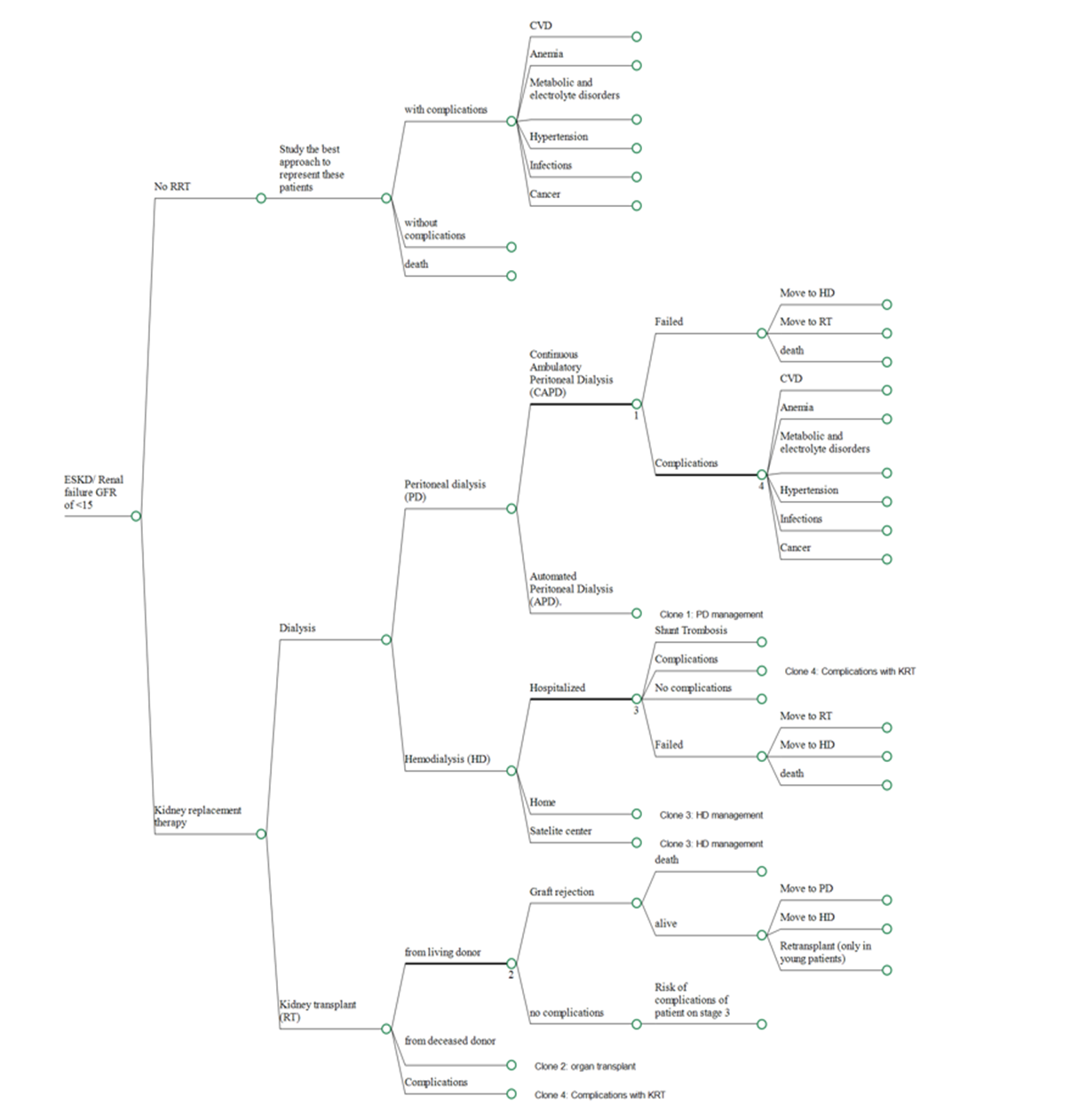


**Figure 2. End-stage kidney disease subtree.**

Abbreviations: CVD = cardiovascular disease; HD, hemodialysis; PD = peritoneal dialysis; KRT = renal replacement therapy; RT, renal transplantation.


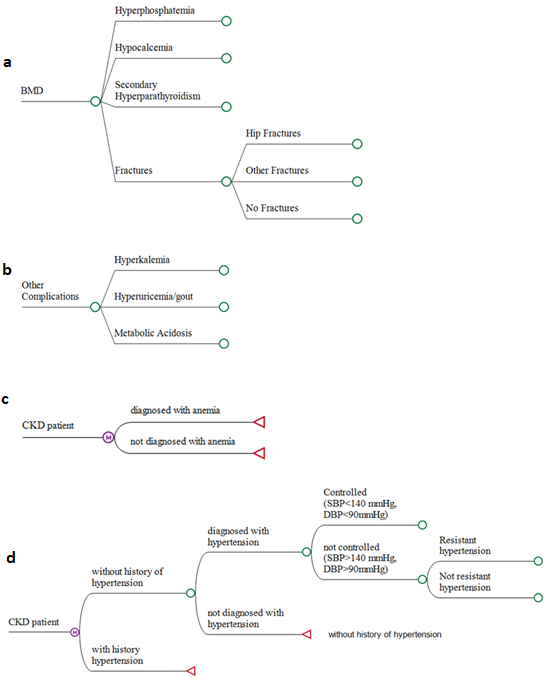


**Figure 3. Bone and mineral disorder and other complications submodels.** a: bone and mineral disorders; b: other complications; c: anemia; d: hypertension.

Abbreviations: BMD = bone and mineral disorder; CKD = chronic kidney disease; DBP = diastolic blood pressure; SBP = systolic blood pressure.


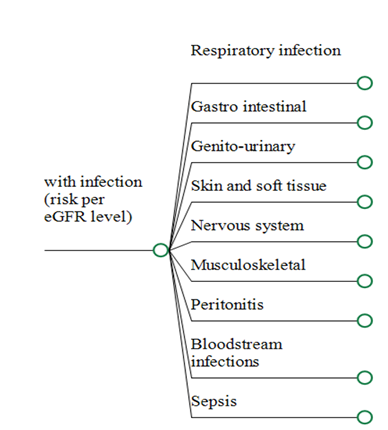


**Figure 4. Infections submodel.**

Abbreviations: eGFR, estimated glomerular filtration rate.


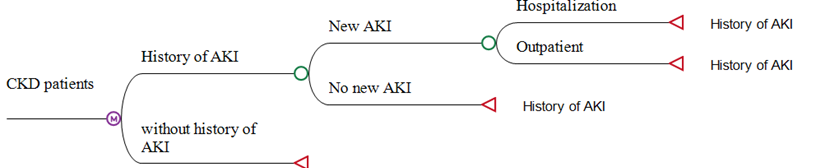


**Figure 5. AKI submodel.**

Abbreviation: AKI, acute kidney injury.


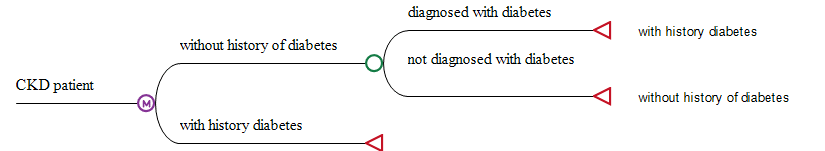


**Figure 6. Diabetes submodel.**

Abbreviation: CKD = chronic kidney disease.

# **Natural disease course modelling**

The natural disease course (without treatment effect) was modelled by the progression of eGFR, uACR, and other risk factors such as HbA1c, BMI, systolic blood pressure, and lipid profile. Based on the disease progression in terms of eGFR, uACR, and other factors, the risk of complications in the sub-models was updated every cycle.

## **eGFR progression**

Annual changes in eGFR in CKD patients with and without diabetes as reported by Grams et al. [1] were used in the model. The value applied in the next cycle of the model, depends on the patient’s KDIGO class in the previous cycle of the simulation. Previous eGFR value and KDIGO-specific slope are summed to generate the new eGFR in the next cycle (Table S1). As no standard deviation was reported, a variation of 10% was applied on these values.

**Table S1. KDIGO-specific slopes used to model eGFR progression in the model.** [1]

| eGFR annual slope | Patients with Diabetes | | | Patients without Diabetes | | |
| --- | --- | --- | --- | --- | --- | --- |
|  | **A1** | **A2** | **A3** | **A1** | **A2** | **A3** |
| G2 | -0.8 | -2.2 | -4.6 | -0.1 | -1.0 | -3.1 |
| G3a | -0.3 | -2.1 | -4.6 | -0.2 | -1.5 | -4.0 |
| G3b | -0.3 | -1.5 | -4.5 | -0.2 | -1.4 | -3.2 |
| G4/5 | -0.1 | -1.1 | -3.6 | -0.2 | -1.2 | -2.8 |

Abbreviation: eGFR = estimated glomerular filtration rate.

## **uACR progression**

Data from Coresh et al. [2] was used to model the uACR progression over time in CKD patients. The distribution of patients per 3-year ACR fold change was extracted from the histogram in Figure S7 and fitted to a lognormal distribution. This distribution allowed for sampling and quantifying the ACR fold change typical of a CKD population. The changes measured by Coresh 2019 [2] (Figure S8), are determined comparing the 3-year values versus baseline assuming that the evidence collected by this research could also be applied at other time points. These random uACR changes were transformed to annual changes, using the cubic root of the value to be applied in annual cycles. Each annual uACR fold change is then multiplied by the uACR value of the previous cycle to obtain the uACR value of the ongoing cycle. It was assumed that the effect of the time-variant exposure equals the effect of the time-invariant exposure. The lognormal distribution applied in the engine has a mean and a standard deviation of 1.0 and 0.93, respectively (visual fitting).


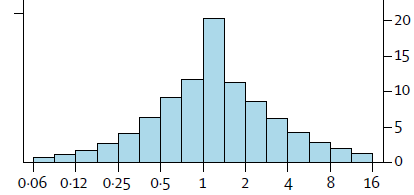


**Figure 7. Histogram of patients of the CKD-PC cohort per 3-year ACR fold change from baseline (log scale)** [2]

Abbreviation: uACR: urine albumin-to-creatinine ratio

x-axis: uACR 3-year fold-change on a lognormal scale; y-axis: percentage of the population


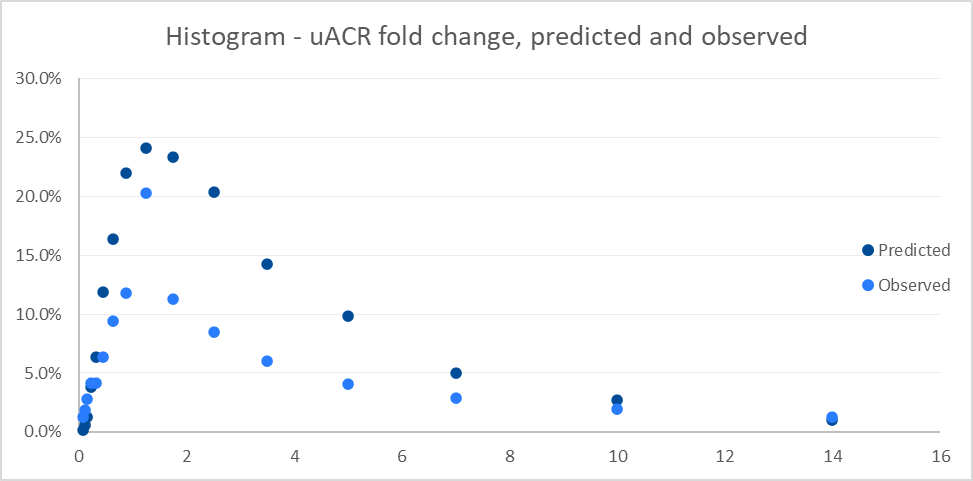


**Figure 8 Distribution of patients per uACR fold change over 3 years (standard scale).**

x-axis: uACR 1-year fold-change on a standard scale; y-axis: percentage of the population

## **Progression of other risk factors**

For the progression of the other risk factors such as HbA1c, lipids, blood pressure and BMI, risk progression engines from the Framingham Heart Study [3] and United Kingdom Prospective Diabetes Study (UKPDS) 90 [4] were used. The equations from the Framingham Heart Study [3] were used for total cholesterol (TC), high-density lipoprotein (HDL), and systolic blood pressure (SBP) and were applied up to 70 years and no further progression is assumed after that (Table S2). Equations from the UKPDS 90 were used to model HbA1c over time in patients with diabetes [4] (Table S3). For patients without diabetes HbA1c progression was programmed using Framingham Offspring study data published by Pani et al. [5] (Table S4). For these individuals it BMI was considered to progress following a constant natural increase in body weight of 0.296 kg per year [6]. This increase was applied until the age of 66 years after which a gradual decrease in the body weight was modelled [7], which was assumed to decrease again with the same slope. BMI progression was calculated using these parameters with the assumption that the height of the patients remained constant over time.

**Table 2. Coefficients of the Framingham risk progression equations for TC, HDL, SBP.** [8]

|  | **TC (mg/dL)** | | **HDL (mg/dL)** | | **SBP (****mm Hg)** | |
| --- | --- | --- | --- | --- | --- | --- |
| **Mean coefficients** | **Males** | **Females** | **Males** | **Females** | **Males** | **Females** |
| Age^1 | -1.48310 | -0.55890 | -0.03900 | 0.00790 | 0.08740 | 0.43750 |
| Age^2 | 0.08450 | 0.02640 | 0.00110 | 0.00040 | -0.00660 | -0.02680 |
| Age^3 | -0.00080 | - | - | - | 0.00020 | 0.00040 |

Abbreviations: HDL = high-density lipoprotein; SBP = systolic blood pressure; TC = total cholesterol

**Table 3. Progression of HbA1c and BMI in patients with diabetes taken from UKPDS 90.** [4]

| **Risk factor (Y)** |  | **HbA1c** | **BMI** |
| --- | --- | --- | --- |
| **Parameters** | **Estimate of coefficient (SE)** | **(%)** | **(kg/m^2^)** |
| Constant | Mean | 1.419 | 0.830 |
|  | SE | 0.041 | 0.039 |
| Female | Mean | 0.054 | 0.045 |
|  | SE | 0.012 | 0.011 |
| African Caribbean | Mean | 0.066 | -0.094 |
|  | SE | 0.026 | 0.016 |
| Asian-Indian | Mean | 0.046 | -0.087 |
|  | SE | 0.020 | 0.014 |
| Value of Y in previous year* | Mean | 0.724 | 0.952 |
|  | SE | 0.005 | 0.003 |
| ln (year since diabetes diagnosis) | Mean | 0.141 | -0.165 |
|  | SE | 0.007 | 0.006 |
| First recorded value of Y | Mean | 0.081 | 0.034 |
|  | SE | 0.007 | 0.003 |

* Three-year lag of Y for risk factors collected every three years
Abbreviations: BMI = body mass index; Hb1Ac = glycaeted haemoglobin; HDL = high-density lipoprotein; SBP = systolic blood pressure; SE = standard errors.

**Table 4. Annual progression of HbA1c in patients without diabetes taken from the Framingham Offspring Study** [5]

| **Age at examination 5 (years)** | **No diabetes** | | | **NGT** | | |
| --- | --- | --- | --- | --- | --- | --- |
|  | **n** | **Mean (%)** | **SE** | **n** | **Mean (%)** | **SE** |
| <40 | 104 | 0.027 | 0.006 | 87 | 0.028 | 0.007 |
| 40–44 | 182 | 0.032 | 0.005 | 153 | 0.026 | 0.006 |
| 45–49 | 337 | 0.037 | 0.004 | 253 | 0.037 | 0.004 |
| 50–54 | 343 | 0.043 | 0.005 | 238 | 0.045 | 0.007 |
| 55–59 | 258 | 0.024 | 0.005 | 165 | 0.02 | 0.006 |
| 60–64 | 239 | 0.024 | 0.006 | 144 | 0.025 | 0.007 |
| 65–69 | 184 | 0.03 | 0.005 | 98 | 0.031 | 0.007 |
| ≥70 | 100 | 0.026 | 0.007 | 59 | 0.024 | 0.009 |

Abbreviations : NGT = Normal glucose tolerance ;SE = Standard error

# **Modelling of events upon disease progression**

### **CVD risk**

The occurrence of the first composite atherosclerotic events were predicted using the risk engine from Matsushita et al. [9] and was subsequently distributed into stroke, coronary heart disease (CHD), myocardial infarction (MI), and angina according to the Framingham population [10,11]. Recurrent atherosclerotic events were predicted using the Framingham recurrent event data [12]. Only one CVD event (MI, stroke, transient ischaemic attack (TIA), angina) could occur per cycle. The risk of hospitalisation due to peripheral artery disease was based on another study by Matsushita et al. [13] that quantified the association of eGFR and uACR with the incidence of PAD-related hospitalisation. The risk of hospitalisation due to heart failure was taken from Grams et al. [1].

### **ESKD risk**

The risk of KRT initiation was based on pooled 5-year data from Tangri et al. [14], and was only applied to patients with an eGFR below 15 ml/min/1.73m2. Patients initiating KRT, were assumed to have a flat eGFR progression. The distribution between the type of KRT (peritoneal dialysis, haemodialysis, and kidney transplant) in the ESKD sub-model were informed by the 2021 report from the UK Renal Registry [15]. Kidney transplantation was only performed in patients under 80 years of age. After initiation of KRT, patients were assumed to discontinue treatment and no further treatment effects were taken into account. It is assumed that patients having a successful transplant move to the health state G3a A1.

### **Other complication risks**

Risks of bone and mineral disorders, anemia, metabolic acidosis and gout stratified by eGFR and were based on various sources: Moranne et al. [16] for hyperparathyroidism, anemia, metabolic acidosis, hyperkalemia, and hyperphosphatemia; Levin et al. [17] for hypocalcemia; Jiaojiao Jing et al. [18] for gout; Vestergaard et al. [19] for anemia; and Runesson et al. [20] for fractures. Diabetes risks were based on validated and updated QDiabetes-2018 prediction algorithms to predict the risk of T2DM for individuals with normal glucose tolerance and with prediabetes [21]. The risk of hypertension was included in the model based on the eGFR level [22]. Patients with KRT are at increased risk of infections. The incidence of infections (respiratory tract, gastrointestinal tract, urinary tract, skin and soft tissue, nervous system, musculoskeletal system, and sepsis) was included in the model based on eGFR levels based on findings of the SCREAM project [23]. The risk of AKI was taken from Sawhney et al. [24]. They estimated that the incidence of AKI in the UK as 1.5% and found an annual rate of 150 events per 10,000 people. In the model, this risk was applied as the reference case to predict the risk of AKI per KDIGO class, using the evidence produced by James et al. [25]. The risk of renal and urothelial cancer was included in the model based on eGFR level using data from a retrospective cohort study in 1.2 million adults [26].

# **References**

1. Grams ME, Surapaneni A, Appel LJ, Lash JP, Hsu J, Diamantidis CJ, et al. Clinical events and patient-reported outcome measures during CKD progression: findings from the Chronic Renal Insufficiency Cohort Study. Nephrol Dial Transplant Off Publ Eur Dial Transpl Assoc - Eur Ren Assoc. 2021;36:1685–93.

2. Coresh J, Heerspink HJL, Sang Y, Matsushita K, Arnlov J, Astor BC, et al. Change in albuminuria and subsequent risk of end-stage kidney disease: an individual participant-level consortium meta-analysis of observational studies. Lancet Diabetes Endocrinol. 2019;7:115–27.

3. Wang W, Bhole VM, Krishnan E. Chronic kidney disease as a risk factor for incident gout among men and women: retrospective cohort study using data from the Framingham Heart Study. BMJ Open. 2015;5:e006843–e006843.

4. Pugh D, Gallacher PJ, Dhaun N. Management of Hypertension in Chronic Kidney Disease. Drugs. 2019;79:365–79.

5. Pani LN, Korenda L, Meigs JB, Driver C, Chamany S, Fox CS, et al. Effect of aging on A1C levels in individuals without diabetes: evidence from the Framingham Offspring Study and the National Health and Nutrition Examination Survey 2001-2004. Diabetes Care. 2008;31:1991–6.

6. Iyen B, Weng S, Vinogradova Y, Akyea RK, Qureshi N, Kai J. Long-term body mass index changes in overweight and obese adults and the risk of heart failure, cardiovascular disease and mortality: a cohort study of over 260,000 adults in the UK. BMC Public Health. 2021;21:576.

7. Zaninotto P, Lassale C. Socioeconomic trajectories of body mass index and waist circumference: results from the English Longitudinal Study of Ageing. BMJ Open. 2019;9:e025309.

8. Wilson PW, Evans JC. Coronary artery disease prediction. Am J Hypertens. 1993;6:309S-313S.

9. Matsushita K, Jassal SK, Sang Y, Ballew SH, Grams ME, Surapaneni A, et al. Incorporating kidney disease measures into cardiovascular risk prediction: Development and validation in 9 million adults from 72 datasets. EClinicalMedicine. 2020;27:100552.

10. D’Agostino RB, Russell MW, Huse DM, Ellison RC, Silbershatz H, Wilson PW, et al. Primary and subsequent coronary risk appraisal: new results from the Framingham study. Am Heart J. 2000;139:272–81.

11. Wolf PA, D’Agostino RB, Belanger AJ, Kannel WB. Probability of stroke: a risk profile from the Framingham Study. Stroke. 1991;22:312–8.

12. Eriksson SE, Olsson JE. Survival and recurrent strokes in patients with different subtypes of stroke: a fourteen-year follow-up study. Cerebrovasc Dis Basel Switz. 2001;12:171–80.

13. Matsushita K, Ballew SH, Coresh J, Arima H, Ärnlöv J, Cirillo M, et al. Measures of chronic kidney disease and risk of incident peripheral artery disease: a collaborative meta-analysis of individual participant data. Lancet Diabetes Endocrinol. 2017;5:718–28.

14. Tangri N, Grams ME, Levey AS, Coresh J, Appel LJ, Astor BC, et al. Multinational Assessment of Accuracy of Equations for Predicting Risk of Kidney Failure: A Meta-analysis. JAMA. 2016;315:164–74.

15. | The UK Kidney Associatio. UKRR. 24th UKRR Annual Report. [Internet]. [cited 2023 Dec 15]. Available from: https://ukkidney.org/audit-research/annual-report/24th-annual-report-data-31122020.

16. Moranne O, Froissart M, Rossert J, Gauci C, Boffa J-J, Haymann JP, et al. Timing of onset of CKD-related metabolic complications. J Am Soc Nephrol JASN. 2009;20:164–71.

17. Levin A, Bakris GL, Molitch M, Smulders M, Tian J, Williams LA, et al. Prevalence of abnormal serum vitamin D, PTH, calcium, and phosphorus in patients with chronic kidney disease: Results of the study to evaluate early kidney disease. Kidney Int. 2007;71:31–8.

18. Jing J, Kielstein JT, Schultheiss UT, Sitter T, Titze SI, Schaeffner ES, et al. Prevalence and correlates of gout in a large cohort of patients with chronic kidney disease: the German Chronic Kidney Disease (GCKD) study. Nephrol Dial Transplant Off Publ Eur Dial Transpl Assoc - Eur Ren Assoc. 2015;30:613–21.

19. Vestergaard SV, Heide-Jørgensen U, van Haalen H, James G, Hedman K, Birn H, et al. Risk of Anemia in Patients with Newly Identified Chronic Kidney Disease - A Population-Based Cohort Study. Clin Epidemiol. 2020;12:953–62.

20. Runesson B, Trevisan M, Iseri K, Qureshi AR, Lindholm B, Barany P, et al. Fractures and their sequelae in non-dialysis-dependent chronic kidney disease: the Stockholm CREAtinine Measurement project. Nephrol Dial Transplant Off Publ Eur Dial Transpl Assoc - Eur Ren Assoc. 2020;35:1908–15.

21. Hippisley-Cox J, Coupland C. Development and validation of QDiabetes-2018 risk prediction algorithm to estimate future risk of type 2 diabetes: cohort study. BMJ. 2017;359:j5019.

22. Vidal-Petiot E, Metzger M, Faucon A-L, Boffa J-J, Haymann J-P, Thervet E, et al. Extracellular Fluid Volume Is an Independent Determinant of Uncontrolled and Resistant Hypertension in Chronic Kidney Disease: A NephroTest Cohort Study. J Am Heart Assoc. 2018;7:e010278.

23. Xu H, Gasparini A, Ishigami J, Mzayen K, Su G, Barany P, et al. eGFR and the Risk of Community-Acquired Infections. Clin J Am Soc Nephrol CJASN. 2017;12:1399–408.

24. Sawhney S, Robinson HA, van der Veer SN, Hounkpatin HO, Scale TM, Chess JA, et al. Acute kidney injury in the UK: a replication cohort study of the variation across three regional populations. BMJ Open. 2018;8:e019435.

25. James MT, Grams ME, Woodward M, Elley CR, Green JA, Wheeler DC, et al. A Meta-analysis of the Association of Estimated GFR, Albuminuria, Diabetes Mellitus, and Hypertension With Acute Kidney Injury. Am J Kidney Dis Off J Natl Kidney Found. 2015;66:602–12.

26. Lowrance WT, Ordoñez J, Udaltsova N, Russo P, Go AS. CKD and the risk of incident cancer. J Am Soc Nephrol JASN. 2014;25:2327–34.
