# Supplementary material for: Cost effectiveness of empagliflozin in adult patients with chronic kidney disease in the Netherlands
Source: PLoS One. 2024 Dec 10;19(12):e0315509. doi: 10.1371/journal.pone.0315509 (PMC11630597; doi:10.1371/journal.pone.0315509)
Supplement: S1 Table — (DOCX) [file pone.0315509.s002.docx]

# **Supplementary Materials**

**Cost effectiveness of empagliflozin in adult patients with chronic kidney disease in the Netherlands**

Tanja Fens^1,2^¶ ([0000-0003-3995-447X](https://orcid.org/0000-0003-3995-447X)), Bart P.H. Slob^1,2^*¶ ([0009-0008-9125-0190](https://orcid.org/0009-0008-9125-0190)), Maaike Weersma^3^, Maarten J. Postma ([0000-0002-6306-3653](https://orcid.org/0000-0002-6306-3653))^1,2,4,5,6^, Cornelis Boersma ([0000-0002-1190-2638](https://orcid.org/0000-0002-1190-2638))^1,2,7^ and Lisa de Jong^1,2^ ([0000-0001-8814-0670](https://orcid.org/0000-0001-8814-0670))

1. Department of Health Sciences, University Medical Center Groningen, University of Groningen, The Netherlands
2. Health-Ecore Ltd, Groningen/ Zeist, The Netherlands
3. Boehringer Ingelheim bv, Amsterdam, The Netherlands
4. Department of Economics, Econometrics & Finance, Faculty of Economics & Business, University of Groningen, The Netherlands
5. Department of Pharmacology and Therapy, Faculty of Medicine, Universitas Airlangga, Indonesia
6. Center of Excellence in Higher Education for Pharmaceutical Care Innovation, Universitas Padjadjaran, Indonesia
7. Department of Management Sciences, Open University, Heerlen, The Netherlands

*Corresponding author:

E-mail: [bartslob@health-ecore.com](mailto:bartslob@health-ecore.com) (BS)

¶ These authors contributed equally to this work

**Table S1. Baseline characteristics used in the model.**

| **Parameter** | **Mean (SD)** | **Distribution** | **Source** |
| --- | --- | --- | --- |
| Age, years | 63.3 (13.9) | Truncated normal | EMPA-KIDNEY trial[2] |
| Gender, % men | 66.8 | Binominal |  |
| Race, % |  |  |  |
| Caucasian | 58.4 | Dirichlet | EMPA-KIDNEY trial[2] |
| Black | 4.0 | Dirichlet |  |
| Asian and Indian | 36.2 | Dirichlet |  |
| Hispanic-Caribbean | 1.4 | Dirichlet |  |
| eGFR class distribution, % |  |  |  |
| G1 | 0.0 | Dirichlet | EMPA-KIDNEY trial [2] |
| G2 | 7.7 | Dirichlet |  |
| G3a | 13.4 | Dirichlet |  |
| G3b | 44.3 | Dirichlet |  |
| G4 | 34.5 | Dirichlet |  |
| G5 | 0.0 | Dirichlet |  |
| uACR class distribution, % |  |  |  |
| A1 | 20.1 | Dirichlet | EMPA-KIDNEY trial[2] |
| A2 | 28.2 | Dirichlet |  |
| A3 | 51.7 | Dirichlet |  |
| Clinical risk factors |  |  |  |
| Smoking, % | 44.6 | Binominal | EMPA-KIDNEY trial[2] |
| eGFR, ml/min per 1.73m2 | 37.3 (14.5) | Truncated normal |  |
| uACR, mg/g | 829.1 (1277.0) | Lognormal |  |
| HbA1c, %-point | 6.3 (1.3) | Truncated normal |  |
| BMI, kg/m2 | 29.7 (6.8) | Truncated normal |  |
| TC, mg/dL | 183.0 (44.5) | Truncated normal | Lash et al. 2009 [3] |
| HDL, mg/dL | 48.1 (15.6) | Truncated normal |  |
| SBP, mmHg | 136.5 (18.3) | Truncated normal | EMPA-KIDNEY trial[2] |
| Height, m | 1.7 (1.0) | Truncated normal |  |
| History of comorbidities, % |  |  |  |
| Diabetes | 46.0 | Binominal | EMPA-KIDNEY trial[2] |
| CVD | 26.7 | Binominal |  |
| Hypertension | 86.1 | Binominal | Grams et al. 2020[4] |
| Heart failure | 9.9 | Binominal | EMPA-KIDNEY trial[2] |
| Gestational diabetes | 0.4 | Binominal | Hippisley-Cox et al. 2017[5] |
| Schizophrenia or bipolar affective disorder | 0.8 | Binominal |  |
| Polycystic ovary syndrome | 2.0 | Binominal |  |
| Learning disability | 1.0 | Binominal |  |

Abbreviations: BMI=body mass index, CKD=chronic kidney disease, CVD=cardiovascular disease, eGFR=estimated glomerular filtration rate, HDL=high-density lipoprotein, uACR=urine albumin-creatinine ratio, SBP=systolic blood pressure, SD=standard deviation, TC=total cholesterol

# **References**

1. Sumida K, Molnar MZ, Potukuchi PK, George K, Thomas F, Lu JL, et al. Changes in Albuminuria and Subsequent Risk of Incident Kidney Disease. Clin J Am Soc Nephrol CJASN. 2017;12:1941–9.

2. W.G. Herrington NS C Wanner, JB Green, SJ Hauske, JR Emberson, D Preiss, P Judge, KJ Mayne, SYA Ng, E Sammons, D Zhu, M Hill, W Stevens, K Wallendszus, S Brenner, AK Cheung, ZH Liu, J Li, LS Hooi, W Liu, T Kadowaki, M Nangaku, A Levin, D Cherney, AP Maggioni, R Pontremoli, R Deo, S Goto, X Rossello, KR Tuttle, D Steubl, M Petrini, D Massey, J Eilbracht, M Brueckmann, MJ Landray, C Baigent, and R Haynes. Empagliflozin in Patients with Chronic Kidney Disease. N Engl J Med. 2023;388:117–27.

3. Lash JP, Go AS, Appel LJ, He J, Ojo A, Rahman M, et al. Chronic Renal Insufficiency Cohort (CRIC) Study: baseline characteristics and associations with kidney function. Clin J Am Soc Nephrol CJASN. 2009;4:1302–11.

4. Grams ME, Surapaneni A, Appel LJ, Lash JP, Hsu J, Diamantidis CJ, et al. Clinical events and patient-reported outcome measures during CKD progression: findings from the Chronic Renal Insufficiency Cohort Study. Nephrol Dial Transplant Off Publ Eur Dial Transpl Assoc - Eur Ren Assoc. 2021;36:1685–93.

5. Hippisley-Cox J, Coupland C. Development and validation of QDiabetes-2018 risk prediction algorithm to estimate future risk of type 2 diabetes: cohort study. BMJ. 2017;359:j5019.
