# Supplementary material for: Cost effectiveness of empagliflozin in adult patients with chronic kidney disease in the Netherlands
Source: PLoS One. 2024 Dec 10;19(12):e0315509. doi: 10.1371/journal.pone.0315509 (PMC11630597; doi:10.1371/journal.pone.0315509)
Supplement: S2 Table — (DOCX) [file pone.0315509.s003.docx]

# **Supplementary Materials**

**Cost effectiveness of empagliflozin in adult patients with chronic kidney disease in the Netherlands**

Tanja Fens^1,2^¶ ([0000-0003-3995-447X](https://orcid.org/0000-0003-3995-447X)), Bart P.H. Slob^1,2^*¶ ([0009-0008-9125-0190](https://orcid.org/0009-0008-9125-0190)), Maaike Weersma^3^, Maarten J. Postma ([0000-0002-6306-3653](https://orcid.org/0000-0002-6306-3653))^1,2,4,5,6^, Cornelis Boersma ([0000-0002-1190-2638](https://orcid.org/0000-0002-1190-2638))^1,2,7^ and Lisa de Jong^1,2^ ([0000-0001-8814-0670](https://orcid.org/0000-0001-8814-0670))

1. Department of Health Sciences, University Medical Center Groningen, University of Groningen, The Netherlands
2. Health-Ecore Ltd, Groningen/ Zeist, The Netherlands
3. Boehringer Ingelheim bv, Amsterdam, The Netherlands
4. Department of Economics, Econometrics & Finance, Faculty of Economics & Business, University of Groningen, The Netherlands
5. Department of Pharmacology and Therapy, Faculty of Medicine, Universitas Airlangga, Indonesia
6. Center of Excellence in Higher Education for Pharmaceutical Care Innovation, Universitas Padjadjaran, Indonesia
7. Department of Management Sciences, Open University, Heerlen, The Netherlands

*Corresponding author:

E-mail: [bartslob@health-ecore.com](mailto:bartslob@health-ecore.com) (BS)

¶ These authors contributed equally to this work

**Table S2. Utility values used in the model.**

| **KDIGO classification** | **Mean** | **Lower value** | **Upper value** | **Source** |
| --- | --- | --- | --- | --- |
| G2*A1 | 0.8625 | 0.8565 | 0.8684 | EMPA-KIDNEY trial[1], Versteegh et al. 2016[2] |
| G2*A2 | 0.8625 | 0.8565 | 0.8684 |  |
| G2*A3 | 0.8623 | 0.8549 | 0.8697 |  |
| G3a*A1 | 0.8570 | 0.8519 | 0.8621 |  |
| G3a*A2 | 0.8569 | 0.8533 | 0.8604 |  |
| G3a*A3 | 0.8568 | 0.8515 | 0.8620 |  |
| G3b*A1 | 0.8570 | 0.8519 | 0.8621 |  |
| G3b*A2 | 0.8569 | 0.8533 | 0.8604 |  |
| G3b*A3 | 0.8568 | 0.8515 | 0.8620 |  |
| G4*A1 | 0.8494 | 0.8437 | 0.8551 |  |
| G4*A2 | 0.8493 | 0.8459 | 0.8526 |  |
| G4*A3 | 0.8491 | 0.8448 | 0.8534 |  |
| G5*A1 | 0.7539 | 0.6632 | 0.9765 |  |
| G5*A2 | 0.7539 | 0.6632 | 0.9765 |  |
| G5*A3 | 0.7539 | 0.6632 | 0.9765 |  |
| **ESRD management** | | | | |
| Peritoneal dialysis | 0.5800 | 0.5000 | 0.6700 | Liem et al. 2008 [3] |
| Hemodialysis | 0.5600 | 0.4900 | 0.6200 |  |
| Kidney transplant | 0.8100 | 0.7200 | 0.9000 | National Institute for Care and Excellence, TA775 [4] |

Note: Utilities were varied in the probabilistic sensitivity analysis using a beta distribution.

Abbreviations: A=KDIGO uACR category, ESKD=end-stage kidney disease, G=KDIGO eGFR category, KDIGO=kidney disease: improving global outcomes, TA=technical appraisal, uACR=urine albumin-creatinine ratio

# **References**

1. W.G. Herrington NS C Wanner, JB Green, SJ Hauske, JR Emberson, D Preiss, P Judge, KJ Mayne, SYA Ng, E Sammons, D Zhu, M Hill, W Stevens, K Wallendszus, S Brenner, AK Cheung, ZH Liu, J Li, LS Hooi, W Liu, T Kadowaki, M Nangaku, A Levin, D Cherney, AP Maggioni, R Pontremoli, R Deo, S Goto, X Rossello, KR Tuttle, D Steubl, M Petrini, D Massey, J Eilbracht, M Brueckmann, MJ Landray, C Baigent, and R Haynes. Empagliflozin in Patients with Chronic Kidney Disease. N Engl J Med. 2023;388:117–27.

2. Versteegh MM, Vermeulen KM, Evers SMAA, Wit GA de, Prenger R, Stolk EA. Dutch Tariff for the Five-Level Version of EQ-5D. Value Health. 2016;19:343–52.

3. Liem YS, Bosch JL, Hunink MGM. Preference-based quality of life of patients on renal replacement therapy: a systematic review and meta-analysis. Value Health J Int Soc Pharmacoeconomics Outcomes Res. 2008;11:733–41.

4. Overview | Dapagliflozin for treating chronic kidney disease | Guidance | NICE [Internet]. NICE; 2022 [cited 2024 Feb 2]. Available from: https://www.nice.org.uk/guidance/ta775
