# Supplementary material for: Cost effectiveness of empagliflozin in adult patients with chronic kidney disease in the Netherlands
Source: PLoS One. 2024 Dec 10;19(12):e0315509. doi: 10.1371/journal.pone.0315509 (PMC11630597; doi:10.1371/journal.pone.0315509)
Supplement: S3 Table — (DOCX) [file pone.0315509.s004.docx]

# **Supplementary Materials**

**Cost effectiveness of empagliflozin in adult patients with chronic kidney disease in the Netherlands**

Tanja Fens^1,2^¶ ([0000-0003-3995-447X](https://orcid.org/0000-0003-3995-447X)), Bart P.H. Slob^1,2^*¶ ([0009-0008-9125-0190](https://orcid.org/0009-0008-9125-0190)), Maaike Weersma^3^, Maarten J. Postma ([0000-0002-6306-3653](https://orcid.org/0000-0002-6306-3653))^1,2,4,5,6^, Cornelis Boersma ([0000-0002-1190-2638](https://orcid.org/0000-0002-1190-2638))^1,2,7^ and Lisa de Jong^1,2^ ([0000-0001-8814-0670](https://orcid.org/0000-0001-8814-0670))

1. Department of Health Sciences, University Medical Center Groningen, University of Groningen, The Netherlands
2. Health-Ecore Ltd, Groningen/ Zeist, The Netherlands
3. Boehringer Ingelheim bv, Amsterdam, The Netherlands
4. Department of Economics, Econometrics & Finance, Faculty of Economics & Business, University of Groningen, The Netherlands
5. Department of Pharmacology and Therapy, Faculty of Medicine, Universitas Airlangga, Indonesia
6. Center of Excellence in Higher Education for Pharmaceutical Care Innovation, Universitas Padjadjaran, Indonesia
7. Department of Management Sciences, Open University, Heerlen, The Netherlands

*Corresponding author:

E-mail: [bartslob@health-ecore.com](mailto:bartslob@health-ecore.com) (BS)

¶ These authors contributed equally to this work

**Table S3. Cost inputs used in the model (EUR, 2022).**

| **Cost parameter** | **Cost** (€) | **SE** | **Source** |
| --- | --- | --- | --- |
| **Treatment costs** | | | |
| Empagliflozin 10 mg | 511.31 | Fixed | Z-index October 2024 [1]. List price including VAT |
| SoC^a^ | 45.17 | Fixed |  |
| **Management cost per health state** | | | |
| G2*A1 | 1,550 | 387.40 | Pollock et al. 2022[2] |
| G2*A2 | 1,941 | 485.37 |  |
| G2*A3 | 2,533 | 633.18 |  |
| G3a*A1 | 1,594 | 398.40 |  |
| G3a*A2 | 1,884 | 470.93 |  |
| G3a*A3 | 2,480 | 620.12 |  |
| G3b*A1 | 1,841 | 460.27 |  |
| G3b*A2 | 2,174 | 543.46 |  |
| G3b*A3 | 3,014 | 753.49 |  |
| G4*A1 | 2,310 | 577.49 |  |
| G4*A2 | 2,707 | 676.84 |  |
| G4*A3 | 3,641 | 910.24 |  |
| G5*A1 | 2,610 | 652.43 |  |
| G5*A2 | 3,191 | 797.83 |  |
| G5*A3 | 6,009 | 1,502 |  |
| **Event cost** | | | |
| ***CVD*** | | | |
| MI IY/ SY | 6,419/ 1,236 | 1,605/ 308.97 | National Health Care Institute Dossier SGLT2 inhibitors in diabetes type 2[3], Soekhal et al. 2013[4] |
| Unstable angina IY/ SY | 6,419 /549.86 | 1,605/ 134.97 |  |
| Stroke IY/ SY | 6,812/2,370 | 1,703/ 592.53 | National Health Care Institute Dossier SGLT2 inhibitors in diabetes type 2[3], Buisman et al. 2015[5] |
| Congestive heart failure (CHF) (chronic) IY/ SY | 13,074/ 1,149 | 3,268/ 287.23 | National Health Care Institute Dossier SGLT2 inhibitors in diabetes type 2[3], Postmus et al. 2011[6] |
| Transient Ischemic Attack (TIA) IY/ SY | 3,158/ 1,610 | 789.38/402.49 | National Health Care Institute Dossier SGLT2 inhibitors in diabetes type 2[3], Buisman et al. 2015[5] |
| Peripheral Artery Disease (PAD) IY/ SY | 2,813/ 521.49 | 703.30/130.37 | Ministry of Health, Welfare and Sport 2016[7] |
| ***ESKD*** | | | |
| Conservative Therapy | 7,097 | 1,774 | Verberne et al. 2018[8] |
| Continuous ambulatory peritoneal dialysis (CAPD) | 94,765 | 23,691 | Mohnen et al. 2019[9] |
| % patients on CAPD | 0.51 | 0.13 |  |
| Automated peritoneal dialysis (APD) | 109,873 | 27,468 |  |
| % patients on APD | 0.49 | 0.12 |  |
| Hemodialysis | 106,353 | 26,588 |  |
| Kidney transplant (living donor) | 89,646 | 22,412 |  |
| Kidney transplant (deceased donor) | 121,502 | 30,375 |  |
| % of patients getting living donor renal transplant | 0.54 | 0.13 |  |
| AKI - outpatient | 333.26 | 83.32 | National Health Care Institute Dossier SGLT2 inhibitors in diabetes type 2[3] |
| AKI - hospitalization | 5,180 | 1,295 |  |
| Peritonitis | 108,616 | 27,154 | Opmeer BC et al. 2010[10] |
| AV access thrombosis | 12,277 | 3,069 | Medical Specialists Federation 2024[11] |
| Bloodstream infections | 12,230 | 3,057 | Manoukian et al. 2021[12] |
| Immunosuppressive Therapy for kidney transplantation - SY | 7,872 | 1,968 | van den Brand et al. 2017[13] |
| ***Metabolic and mineral disorder*** | | | |
| Metabolic Acidosis | 1,047 | 261.81 | National Health Care Institute Dossier empagliflozine (Jardiance®)[14] |
| Hyperkalemia | 145.73 | 36.43 |  |
| Hyperphosphatemia | 1017 | 254.17 | British National Formulary[15] |
| Hyperparathyroidism | 606.3 | 151.58 | Dutch Healthcare Authority (NZa) Open DIS database- weighted average of code 0313-231 [16] |
| Hyperuricemia/gout | 7,171 | 1,793 | Spaetgens et al. 2015[17] |
| Hypocalcemia | 1,017 | 254.30 | British National Formulary[15] |
| ***Bone and skeleton disorders*** | | | |
| Hip fractures | 28,892 | 7,223 | Hartholt et al. 2012[18] |
| Other fractures | 7,671 | 1,918 |  |
| ***Infections*** | | | |
| Respiratory infections | 156.93 | 39.23 | Kohli et al. 2021[19] |
| Urinary tract infection | 26.78 | 6.70 | National Health Care Institute Dossier empagliflozine (Jardiance®)[14] |
| Skin and soft tissue infections | 2,383 | 595.68 | Dutch Healthcare Authority (NZa) DIS -weighted average of code 0313-491[16] |
| Gastrointestinal infection | 290 | 72.5 | Dutch Healthcare Authority (NZa) DIS- health product 119499078[16] |
| Muscular infections | 5,763 | 1,441 | NHS England 2020/21[20] |
| Nervous system | 1,740 | 434,94 | Dutch Healthcare Authority (NZa) DIS -weighted average of code 0330-0811[16] |
| Sepsis | 31,013 | 7,753 | Brouwer et al. 2019[21] |
| Anemia | 1,931 | 482.68 | National Institute for Health and Care and Excellence (NICE) 2017, TA780[22] |
| ***Cancer*** | | | |
| Renal cancer | 15,769 | 3,942 | Amdahl et al. 2017[23] |
| Urothelial cancer | 16,680 | 4,170 | Sangar et al. 2005[24] |
| All cause Hospitalizations IY/ SY | 608.33/ 541.13 | 152.08/135.28 | Dutch cost manual[25] |
| ***Adverse events (lower limb amputations)*** | | | |
| Leg amputation | 19,575 | 4,894 | Van Schoonhoven et al. 2023[26] |
| Toe amputation | 9,788 | 2,447 | Assumption |
| Foot amputation | 9,788 | 2,447 | Assumption |
| **Societal costs** | | | |
| ***Informal care*** | | | |
| G2*A1-A3 | - | - | Assumption |
| G3a*A1-A3 | 853.42 | 213.36 | Turchetti et al 2017[27]/Elshahat et al 2020[28] |
| G3b*A1-A3 | 853.42 | 213.36 | Turchetti et al 2017[27] |
| G4*A1-A3 | 2,385 | 596.25 | Turchetti et al 2017[27] |
| G5*A1-A3 | 2,310 | 577.50 | De Vries et al 2021[29] |
| Kidney transplantation | 2,362 | 590.50 | De Vries et al 2021[29] |
| Dialysis | 7,505 | 1,876 | Turchetti et al 2017[27]/ Elshahat et al 2020[28] |
| ***Travel costs^b^*** |  |  |  |
| G2*A1-A3 | 16.16 | 4.04 | National Health Care Institute, Dossier dapagliflozin CKD [30] ^d^ |
| G3a*A1-A3 | 59.82 | 14.96 | National Health Care Institute, Dossier dapagliflozin CKD [30] ^e^ |
| G3b*A1-A3 | 59.82 | 14.96 | Turchetti et al 2017[27] |
| G4*A1-A3 | 73.35 | 18.34 | Turchetti et al 2017[27] |
| G5*A1-A3 | 91.69 | 22.92 | Baker et al 2017[31] |
| Kidney transplantation | 194.70 | 48.68 | Federation Medical Specialists[32] ^f^ |
| Dialysis | 1,151 | 287.75 | National Health Care Institute, Dossier dapagliflozin CKD [30] |
| ***Productivity losses^g^*** |  |  |  |
| G2*A1-A3 | - | - | Assumption |
| G3a*A1-A3 | 20.35 | 5.09 | Elshahat et al 2020[28]/ Turchetti et al 2017[27] |
| G3b*A1-A3 | 20.35 | 5.09 | Turchetti et al 2017[27] |
| G4*A1-A3 | 35.72 | 8.93 | Turchetti et al 2017[27] |
| G5*A1-A3 | 76.22 | 19.06 | De Vries et al 2021[29] |
| Kidney transplantation | 521.16 | 130.29 | De Vries et al 2021[29] |
| Dialysis | 1,451 | 362.75 | Elshahat et al 2020[28]/ Turchetti et al 2017[27] |

| Note: Costs were varied in the probabilistic sensitivity analysis using a gamma distribution.  ^a^ Weighted average based on the concomitant medication use of renin-angiotensin-system inhibitors in the EMPA-KIDNEY trial[33].  ^b^ The average distances to a general practitioner and hospital were 1.1 km and 7 km, respectively, and a cost per km of €0.21[34].  ^c^ Assuming one GP and one hospital visit per year  ^d^ Assuming one GP and two hospital visits per year  ^e^ Assuming on average 11.6 GP and three hospital visits per year  ^f^ Assuming on average 3.5 dialysis visits per week  ^g^ Corrected for the remaining duration from the median starting age of the cohort to retirement and a work participation rate of individuals aged between 60 and 70 years in the Netherlands (44.25%)[35]  Abbreviations: AKI=Acute kidney injury; IY=initial year, MI=myocardial infarction; SY=subsequent year, SoC=standard of care |
| --- |

# **References**

1. Z-Index [Internet]. [cited 2024 Jan 26]. Available from: https://www.z-index.nl/

2. Pollock C, James G, Garcia Sanchez JJ, Carrero JJ, Arnold M, Lam CSP, et al. Healthcare resource utilisation and related costs of patients with CKD from the UK: a report from the DISCOVER CKD retrospective cohort. Clin Kidney J. 2022;15:2124–34.

3. National Health Care Institute. Farmacotherapeutisch rapport SGLT2- remmers dapa-, cana-, empa- en ertugliflozine (Forxiga®, Invokana®, Jardiance® en Steglatro®) bij de behandeling van volwassenen met diabetes mellitus type 2 met een zeer hoog risico op hart- en vaatziekten [Internet]. 2021. Available from: https://www.zorginstituutnederland.nl/publicaties/adviezen/2021/06/22/gvs-advies-sglt-2-remmers-uitbreiding-bijlage-2

4. Soekhlal RR, Burgers LT, Redekop WK, Tan SS. Treatment costs of acute myocardial infarction in the Netherlands. Neth Heart J Mon J Neth Soc Cardiol Neth Heart Found. 2013;21:230–5.

5. Buisman LR, Tan SS, Nederkoorn PJ, Koudstaal PJ, Redekop WK. Hospital costs of ischemic stroke and TIA in the Netherlands. Neurology. 2015;84:2208–15.

6. Postmus D, Pari AAA, Jaarsma T, Luttik ML, van Veldhuisen DJ, Hillege HL, et al. A trial-based economic evaluation of 2 nurse-led disease management programs in heart failure. Am Heart J. 2011;162:1096–104.

7. National Health Care Institute W en S. Zinnige Zorg - Verbetersignalement Perifeer arterieel vaatlijden (PAV) - Rapport - Zorginstituut Nederland [Internet]. Ministerie van Volksgezondheid, Welzijn en Sport; 2016 [cited 2024 Jan 26]. Available from: https://www.zorginstituutnederland.nl/publicaties/rapport/2016/08/16/zinnige-zorg-verbetersignalement-perifeer-arterieel-vaatlijden---claudicatio-intermittens

8. Verberne WR, Dijkers J, Kelder JC, Geers ABM, Jellema WT, Vincent HH, et al. Value-based evaluation of dialysis versus conservative care in older patients with advanced chronic kidney disease: a cohort study. BMC Nephrol. 2018;19:205.

9. Mohnen SM, van Oosten MJM, Los J, Leegte MJH, Jager KJ, Hemmelder MH, et al. Healthcare costs of patients on different renal replacement modalities – Analysis of Dutch health insurance claims data. PLoS ONE. 2019;14:e0220800.

10. Opmeer BC, Boer KR, van Ruler O, Reitsma JB, Gooszen HG, de Graaf PW, et al. Costs of relaparotomy on-demand versus planned relaparotomy in patients with severe peritonitis: an economic evaluation within a randomized controlled trial. Crit Care Lond Engl. 2010;14:R97.

11. Federatie Medisch Specialisten. Richtlijn- Vaattoegang voor hemodialyse [Internet]. 2024. Available from: https://richtlijnendatabase.nl/richtlijn/vaattoegang_voor_hemodialyse/operatieve_besluitvorming_bij_vaattoegang_voor_hemodialyse/soort_vaattoegang_en_locatie_voor_hemodialyse.html

12. Manoukian S, Stewart S, Graves N, Mason H, Robertson C, Kennedy S, et al. Bed-days and costs associated with the inpatient burden of healthcare-associated infection in the UK. J Hosp Infect. 2021;114:43–50.

13. Jan AJG van den Brand, Jacobien C Verhave, Eddy M Adang, Jack FM Wetzels. Cost-effectiveness of eculizumab treatment after kidney transplantation in patients with atypical haemolytic uraemic syndrome. Nephrology Dialysis Transplantation. 2017;32:i115–22.

14. National Health Care Institute W en S. GVS-advies empagliflozine (Jardiance®) uitbreiding bijlage 2-voorwaarden voor chronische nierschade - Advies - Zorginstituut Nederland [Internet]. Ministerie van Volksgezondheid, Welzijn en Sport; 2023 [cited 2024 Jan 12]. Available from: https://www.zorginstituutnederland.nl/publicaties/adviezen/2023/12/07/gvs-advies-empagliflozine-jardiance-uitbreiding-bijlage-2-voorwaarden-voor-chronische-nierschade

15. BNF 82 (British National Formulary) 2022 pdf free download [Internet]. Book Med. 2022 [cited 2024 Jan 26]. Available from: https://www.booksofmedical.com/2022/05/bnf-82-british-national-formulary-2022.html

16. Dutch Healthcare Authority (NZa). Open DIS data | Data overheid [Internet]. [cited 2024 Jan 26]. Available from: https://data.overheid.nl/en/dataset/open-dis-data

17. Spaetgens B, Wijnands JMA, van Durme C, van der Linden S, Boonen A. Cost of illness and determinants of costs among patients with gout. J Rheumatol. 2015;42:335–44.

18. Hartholt KA, Polinder S, Van der Cammen TJM, Panneman MJM, Van der Velde N, Van Lieshout EMM, et al. Costs of falls in an ageing population: A nationwide study from the Netherlands (2007–2009). Injury. 2012;43:1199–203.

19. Kohli MA, Maschio M, Mould-Quevedo JF, Ashraf M, Drummond MF, Weinstein MC. The Cost-Effectiveness of Expanding Vaccination with a Cell-Based Influenza Vaccine to Low Risk Adults Aged 50 to 64 Years in the United Kingdom. Vaccines. 2021;9:598.

20. NHS England » 2020/21 National Cost Collection Data Publication [Internet]. [cited 2024 Jan 26]. Available from: https://www.england.nhs.uk/publication/2020-21-national-cost-collection-data-publication/

21. Brouwer ME. Societal impact of sepsis and inflammation in the ICU and beyond [Internet]. Utrecht University; 2019 [cited 2024 Jan 26]. Available from: https://dspace.library.uu.nl/handle/1874/384909

22. National Institute for Health and Care Excellence. Immunosuppressive therapy for kidney transplant in adults [Internet]. NICE; 2017. Available from: www.nice.org.uk/guidance/ta481

23. Amdahl J, Diaz J, Sharma A, Park J, Chandiwana D, Delea TE. Cost-effectiveness of pazopanib versus sunitinib for metastatic renal cell carcinoma in the United Kingdom. PloS One. 2017;12:e0175920.

24. Sangar VK, Ragavan N, Matanhelia SS, Watson MW, Blades RA. The economic consequences of prostate and bladder cancer in the UK. BJU Int. 2005;95:59–63.

25. Kanters TA, Bouwmans CAM, van der Linden N, Tan SS, Hakkaart-van Roijen L. Update of the Dutch manual for costing studies in health care. Prinja S, editor. PLOS ONE. 2017;12:e0187477.

26. van Schoonhoven AV, Schöttler MH, Serné EH, Schrömbges PPG, Postma MJ, Boersma C. The health and budget impact of sodium-glucose co-transporter-2 inhibitors (SGLT2is) in The Netherlands. J Med Econ. 2023;26:547–53.

27. Turchetti G, Bellelli S, Amato M, Bianchi S, Conti P, Cupisti A, et al. The social cost of chronic kidney disease in Italy. Eur J Health Econ HEPAC Health Econ Prev Care. 2017;18:847–58.

28. Elshahat S, Cockwell P, Maxwell AP, Griffin M, O’Brien T, O’Neill C. The impact of chronic kidney disease on developed countries from a health economics perspective: A systematic scoping review. PloS One. 2020;15:e0230512.

29. de Vries EF, Los J, de Wit GA, Hakkaart-van Roijen L. Patient, family and productivity costs of end-stage renal disease in the Netherlands; exposing non-healthcare related costs. BMC Nephrol. 2021;22:341.

30. National Health Care Institute W en S. GVS-advies uitbreiding bijlage 2-voorwaarden dapagliflozine (Forxiga®) bij de behandeling van chronische nierschade - Advies - Zorginstituut Nederland [Internet]. Ministerie van Volksgezondheid, Welzijn en Sport; 2022 [cited 2024 Feb 2]. Available from: https://www.zorginstituutnederland.nl/publicaties/adviezen/2022/03/28/gvs-advies-dapagliflozine-forxiga-bij-chronische-nierschade

31. Baker R, Jardine A, Andrews P. Renal Association Clinical Practice Guideline on post-operative care of the kidney transplant recipient. Nephron Clin Pract. 2011;118 Suppl 1:c311-347.

32. Beleid en behandeling bij CNS - Richtlijn - Richtlijnendatabase [Internet]. [cited 2024 Jan 26]. Available from: https://richtlijnendatabase.nl/richtlijn/chronische_nierschade_cns/beleid_en_behandeling_bij_cns.html

33. W.G. Herrington NS C Wanner, JB Green, SJ Hauske, JR Emberson, D Preiss, P Judge, KJ Mayne, SYA Ng, E Sammons, D Zhu, M Hill, W Stevens, K Wallendszus, S Brenner, AK Cheung, ZH Liu, J Li, LS Hooi, W Liu, T Kadowaki, M Nangaku, A Levin, D Cherney, AP Maggioni, R Pontremoli, R Deo, S Goto, X Rossello, KR Tuttle, D Steubl, M Petrini, D Massey, J Eilbracht, M Brueckmann, MJ Landray, C Baigent, and R Haynes. Empagliflozin in Patients with Chronic Kidney Disease. N Engl J Med. 2023;388:117–27.

34. National Health Care Institute. Guideline for conducting economic evaluations in health care [Internet]. Ministerie van Volksgezondheid, Welzijn en Sport; 2016 [cited 2023 Dec 15]. Available from: https://www.zorginstituutnederland.nl/publicaties/publicatie/2016/02/29/richtlijn-voor-het-uitvoeren-van-economische-evaluaties-in-de-gezondheidszorg

35. Centraal bureau voor de statistiek. StatLine - Arbeidsdeelname; kerncijfers [Internet]. [cited 2023 Dec 1]. Available from: https://opendata.cbs.nl/#/CBS/nl/dataset/85264NED/table?searchKeywords=arbeidsparticipatie
