# Supplementary material for: Cost effectiveness of empagliflozin in adult patients with chronic kidney disease in the Netherlands
Source: PLoS One. 2024 Dec 10;19(12):e0315509. doi: 10.1371/journal.pone.0315509 (PMC11630597; doi:10.1371/journal.pone.0315509)
Supplement: S4 Table — (DOCX) [file pone.0315509.s005.docx]

# **Supplementary Materials**

**Cost effectiveness of empagliflozin in adult patients with chronic kidney disease in the Netherlands**

Tanja Fens^1,2^¶ ([0000-0003-3995-447X](https://orcid.org/0000-0003-3995-447X)), Bart P.H. Slob^1,2^*¶ ([0009-0008-9125-0190](https://orcid.org/0009-0008-9125-0190)), Maaike Weersma^3^, Maarten J. Postma ([0000-0002-6306-3653](https://orcid.org/0000-0002-6306-3653))^1,2,4,5,6^, Cornelis Boersma ([0000-0002-1190-2638](https://orcid.org/0000-0002-1190-2638))^1,2,7^ and Lisa de Jong^1,2^ ([0000-0001-8814-0670](https://orcid.org/0000-0001-8814-0670))

1. Department of Health Sciences, University Medical Center Groningen, University of Groningen, The Netherlands
2. Health-Ecore Ltd, Groningen/ Zeist, The Netherlands
3. Boehringer Ingelheim bv, Amsterdam, The Netherlands
4. Department of Economics, Econometrics & Finance, Faculty of Economics & Business, University of Groningen, The Netherlands
5. Department of Pharmacology and Therapy, Faculty of Medicine, Universitas Airlangga, Indonesia
6. Center of Excellence in Higher Education for Pharmaceutical Care Innovation, Universitas Padjadjaran, Indonesia
7. Department of Management Sciences, Open University, Heerlen, The Netherlands

*Corresponding author:

E-mail: [bartslob@health-ecore.com](mailto:bartslob@health-ecore.com) (BS)

¶ These authors contributed equally to this work

**Table S4. Incidence of events per sub module for empagliflozin plus SoC versus SoC alone.**

| **Submodule** | **Empagliflozin plus SoC** | **SoC** | **Incremental** |
| --- | --- | --- | --- |
| **CVD complications** | | | |
| 1st ASCVD event | 21.20% | 19.40% | 1.80% |
| 1st MI | 9.30% | 7.90% | 1.40% |
| 1st stroke | 2.40% | 2.50% | -0.10% |
| 1st unstable angina | 8.90% | 8.80% | 0.10% |
| 1st TIA | 1.20% | 1.20% | 0.00% |
| HF new onset only | 25.60% | 24.80% | 0.80% |
| PAD new onset only | 11.10% | 9.80% | 1.30% |
| 3-point MACE | 44.80% | 44.70% | 0.10% |
| Rates of events, per 100 patient-years |  |  |  |
| HF hospitalizations (first and recurrent) | 2.59 | 2.89 | HR, 0.90 |
| PAD hospitalizations (first and recurrent) | 0.98 | 0.96 | HR, 1.01 |
| **Bone and Mineral Disorders** | | | |
| All fractures | 47.10% | 41.40% | 5.70% |
| Hip fractures | 23.30% | 19.80% | 3.50% |
| Other fractures | 33.30% | 28.60% | 4.70% |
| **AKI** | | | |
| All AKI | 40.80% | 40.80% | 0.00% |
| AKI hospitalization | 38.30% | 37.70% | 0.60% |
| AKI outpatient | 3.40% | 3.70% | -0.30% |
| Rates of events, per 100 patient-years |  |  |  |
| All AKI events (first and recurrent) | 3.33 | 3.79 | HR, 0.88 |
| AKI hospitalization | 3.06 | 3.46 | HR, 0.89 |
| AKI outpatient | 0.27 | 0.34 | HR, 0.80 |
| **Hospitalizations** | | | |
| All cause hospitalizations | 87.70% | 85.70% | 2.00% |
| All CVD hospitalizations | 50.50% | 47.60% | 2.90% |
| 1st hospitalization caused by infections | 79.50% | 76.20% | 3.30% |
| Rates of events, per 100 patient-years |  |  |  |
| All cause hospitalizations | 30.78 | 31.55 | HR, 0.98 |
| All CVD hospitalizations | 6.99 | 7.28 | HR, 0.96 |
| 1st hospitalization caused infections | 20.06 | 20.29 | HR, 0.99 |
| **Infections** | | | |
| All infections | 87.60% | 86.00% | 1.60% |
| Respiratory tract | 60.80% | 54.90% | 5.90% |
| Gastrointestinal tract | 27.60% | 23.70% | 3.90% |
| Urinary tract | 66.20% | 62.60% | 3.60% |
| Skin and soft tissue | 41.40% | 38.60% | 2.80% |
| Nervous system | 12.10% | 11.50% | 0.60% |
| Musculoskeletal system | 29.40% | 25.00% | 4.40% |
| Sepsis | 27.80% | 25.00% | 2.80% |
| **ESKD** | | | |
| ESKD defined as eGFR under 15 ml/min per 1.73 m^2^ | 60.10% | 70.80% | -10.70% |
| ESKD patients treated with conservative therapy | 35.70% | 38.70% | -3.00% |
| ESKD defined as initiating KRT | 52.50% | 63.00% | -10.50% |
| Peritoneal dialysis | 11.40% | 15.50% | -4.10% |
| Peritonitis | 6.60% | 8.30% | -1.70% |
| Hemodialysis | 46.20% | 54.20% | -8.00% |
| AV access thrombosis | 24.10% | 30.30% | -6.20% |
| Bloodstream infections | 13.20% | 15.30% | -2.10% |
| Kidney transplant (living or deceased donor) | 12.90% | 17.40% | -4.50% |
| Failed kidney transplant | 0.60% | 0.80% | -0.20% |
| **Cancer** | | | |
| Renal | 0.90% | 0.70% | 0.20% |
| Urothelial | 0.30% | 0.10% | 0.20% |

Abbreviations: AKI=acute kidney injury, ASCVD=atherosclerotic cardiovascular disease; AV= arteriovenous, CVD=cardiovascular disease, ESKD=end-stage kidney disease, HR=heart failure, MI=myocardial Infarction; TIA=transient ischemic attack; HF=heart failure; HR=hazard ratio; PAD=peripheral artery disease, MACE=major adverse cardiovascular events, AKI=acute kidney injury, ESKD=end stage kidney disease, KRT=kidney replacement therapy, SoC=standard of care
