# Supplementary material for: Cost effectiveness of empagliflozin in adult patients with chronic kidney disease in the Netherlands
Source: PLoS One. 2024 Dec 10;19(12):e0315509. doi: 10.1371/journal.pone.0315509 (PMC11630597; doi:10.1371/journal.pone.0315509)
Supplement: S1 Fig — (DOCX) [file pone.0315509.s006.docx]

# **Supplementary Materials**

**Cost effectiveness of empagliflozin in adult patients with chronic kidney disease in the Netherlands**

Tanja Fens^1,2^¶ ([0000-0003-3995-447X](https://orcid.org/0000-0003-3995-447X)), Bart P.H. Slob^1,2^*¶ ([0009-0008-9125-0190](https://orcid.org/0009-0008-9125-0190)), Maaike Weersma^3^, Maarten J. Postma ([0000-0002-6306-3653](https://orcid.org/0000-0002-6306-3653))^1,2,4,5,6^, Cornelis Boersma ([0000-0002-1190-2638](https://orcid.org/0000-0002-1190-2638))^1,2,7^ and Lisa de Jong^1,2^ ([0000-0001-8814-0670](https://orcid.org/0000-0001-8814-0670))

1. Department of Health Sciences, University Medical Center Groningen, University of Groningen, The Netherlands
2. Health-Ecore Ltd, Groningen/ Zeist, The Netherlands
3. Boehringer Ingelheim bv, Amsterdam, The Netherlands
4. Department of Economics, Econometrics & Finance, Faculty of Economics & Business, University of Groningen, The Netherlands
5. Department of Pharmacology and Therapy, Faculty of Medicine, Universitas Airlangga, Indonesia
6. Center of Excellence in Higher Education for Pharmaceutical Care Innovation, Universitas Padjadjaran, Indonesia
7. Department of Management Sciences, Open University, Heerlen, The Netherlands

*Corresponding author:

E-mail: [bartslob@health-ecore.com](mailto:bartslob@health-ecore.com) (BS)

¶ These authors contributed equally to this work

**Figure S1. Cost-effectiveness acceptability curve presenting the results of the probabilistic sensitivity analysis.**

Abbreviations: QALY=quality-adjusted life years, SoC=standard of care
