# Supplementary material for: Cost effectiveness of empagliflozin in adult patients with chronic kidney disease in the Netherlands
Source: PLoS One. 2024 Dec 10;19(12):e0315509. doi: 10.1371/journal.pone.0315509 (PMC11630597; doi:10.1371/journal.pone.0315509)
Supplement: Attachment — Submitted filename: Response to Reviewers.docx [file pone.0315509.s007.docx]

**RESPONSE TO THE EDITORS AND REVIEWERS**

Dear editors,

Dear reviewers,

Thank you for your time and valuable comments regarding our work. Please find our response in italic after each of your comments and see the track changes in the updated manuscript.

On behalf of all authors,

Kind regards,

Bart Slob

**Comments from the Editor:**

**The analysis of the Cost Effectiveness of Empagliflozin in Adult Patients with Chronic Kidney Disease in the Netherlands purports to suggest that the addition of empagliflozin to standard of care medications might be cost saving. Several points have been brought up by the reviewers that do need to be addressed.**

- **One, of course is the influence of industry upon the presentation.**

*Appropriate statement reflecting the influence of industry will be included in the financial disclosure in the submission system, reading as follows:*

*“The study was supported and funded by Boehringer Ingelheim (BI). BI was given the opportunity to review the manuscript for medical and scientific accuracy as well as intellectual property considerations. The funder had no role in the model adaptations, conduct of the analyses, and interpretation of the results, nor in the writing of the manuscript.”*

- **Another is on the price of empagliflozin and whether it is similar in other countries (particularly with respect to those outside of the EU).**

*Regarding the price of empagliflozin across countries, we would like to emphasize that HTA bodies vary according to the sets of national priorities and every country has their own reimbursement and pricing policy. Therefore, straightforward comparisons are not very informative but can certainly give indications when looking into neighboring countries or countries with similar economies and organization of their health system, such as UK.*

*In our analyses we used the cost input of €511.31 (updated in the most reason version of the model and manuscript to October 2024 list prices as reported in the G-standard of the Z-index). Another recent cost-effectiveness study in the UK, by Ramos et al. 2024 (reference 33 in the manuscript), used the price of £476.98 (= €565.51; conversion rate 1.18:1) which is comparable to the price used in our model. Another cost-effectiveness analysis was conducted in Asian countries (Varghese et al., reference 32 in the manuscript). Although the conclusion of these analyses was similar (dominant or cost-effective ICER) the empagliflozin pricing was lower, ranging between €288.55 and €409.08 per year.*

*See below the two new references added to our manuscript supporting our feedback to your comment:*

*Ramos, M., Gerlier, L., Uster, A., Muttram, L., Frankel, A. H., & Lamotte, M. (2024). Cost-effectiveness of empagliflozin as add-on to standard of care for chronic kidney disease management in the United Kingdom. Journal of Medical Economics, (just-accepted), 1-15.*

*Varghese, L., Chang, P. W., Juntarasiripas, S., Pham, H., & Uster, A. (2024). Cost-effectiveness of add-on empagliflozin versus standard of care in management of CKD in Malaysia, Thailand and Vietnam–findings from a modelling study assessing an EMPA-KIDNEY eligible population, using CKD progression model. Journal of Medical Economics, 27(1), 836-848.*

*Taking both studies into consideration, we added this information in the discussion section reading as follows (lines 329-333):*

*“Other recent cost-effectiveness analyses on empagliflozin have been conducted in the UK, Malaysia, Thailand and Vietnam which, like us, reported a dominant (UK, Malaysia, Thailand) or cost-effective (Vietnam) ICERs. Although input parameters were country specific, empagliflozin pricing was comparable to our study with £476,98 (€565,51) vs €546,55 for the UK while prices were somewhat lower in the Asian countries (ranging between €288.55 and €409.08 per year) [32,33].”*

- **Yet another, and perhaps even more important is that not all CKD is the same. A large proportion of CKD involves patients with diabetes. It would be helpful to break down the differences in the value of adding empagliflozin to standard of care with respect to nondiabetic vs diabetic patients, or perhaps those who are underweight vs. those who are overweight. This would give us additional information (e.g J Diabetes Complications 2021 Feb;35(2):107761).**

*We acknowledge indeed that not all CKD is the same, as it is a “heterogeneous population” (line 124), that is why we did a scenario analysis in which we made the distinction between patients with and without diabetes (using the submodule presented in fig. 6 in the supplementary material). The results of this analysis showed in both groups (diabetic and non-diabetic) a dominant ICER, with the non-diabetic subgroup presenting a higher incremental cost saving (scenario 2 and 3):*

*This is now explicitly highlighted in the text describing the results of these scenario analyses (lines 297-300):*

*“All scenarios demonstrate dominant ICERs favoring empagliflozin + SoC over SoC alone, including the subgroup analyses exploring the difference between diabetic and non-diabetic patients (scenarios 2 and 3), with most scenarios showing only a marginal effect on the outcomes.*

*In the discussion section, an additional comment on this was made to emphasize that empagliflozin is cost-effective in both patient with and without diabetes (line 309-310): “Empagliflozin remained highly cost-effective in both diabetic and non-diabetic patients.”*

*Although we conclude that empagliflozin is cost-saving for both diabetic and non-diabetic CKD patients, there were some differences in the findings. Whereas there were more cost-savings in the non-diabetic group, the QALY gain was bigger in the diabetes group. We address this matter in the discussion section in lines 362-368:*

*“For the purpose of this study, we conducted two subgroup analyses based on the diabetic and non-diabetic populations from the EMPA-KIDNEY trial. Although the results were relatively comparable between the groups and both resulted in dominant ICERs, the non-diabetic population seems to favor in terms of cost savings, while the diabetic population favors in terms of QALYs gained. This is consistent with findings from a single-center cross-sectional analysis, which suggests that SGTL2 inhibitors are often prescribed to higher-risk diabetic patients, potentially justifying their higher cost due to significant clinical benefits, particularly in cardiovascular and renal outcomes [38]. ”*

**Comments from the Reviewers:**

**Reviewer #1:**

**This study provides valuable insights into the cost-effectiveness of empagliflozin for patients with chronic kidney disease (CKD) in the Netherlands. It is commendable that the researchers adhered to the KDIGO guidelines for CKD definition, which include both GFR levels and for eGFR >60 ml/min also the evidence of kidney damage, such as albuminuria or structural abnormalities.**

- **The Empa Kidney study inclusion criteria, focusing on patients with eGFR less than 45 ml/min/1.73 m2 or those with eGFR between 45 and 90 ml/min/1.73 m2 and albuminuria, ensured that all participants met the KDIGO criteria. The current study presentation, particularly Figure 1, does not adequately address this aspect.**
- **Additionally, it would be insightful to know if patients with eGFR greater than 60 ml/min without albuminuria or other signs of kidney damage, who would not meet the KDIGO criteria for CKD, were included. Furthermore, data on patients with eGFR between 45 and 60 ml/min, who meet KDIGO criteria but were excluded from the Empa kidney study, would provide valuable context and contribute to a more comprehensive understanding of empagliflozin's cost-effectiveness across different stages of CKD.**

*Very valid points have been made by the reviewer. We acknowledge this needs clarification in the manuscript. Based on the combination of eGFR and uACR levels from the EMPA-Kidney trial, patients were assigned to one of the possible health states at baseline (which is represented by the matrix presented in Figure 1). The disease progression was modelled based on these health states. In all health states the patient can experience the same set of complications, including ESKD, cardiovascular disease, diabetes, hypertension, cancer, bone and mineral disorders, infections, acute kidney injury (AKI), anemia, which were modelled in separate sub-models. However, the risk of these complications was health state-specific and varied based on eGFR, uACR, and other factors such as glycated haemoglobin (HbA1C) and body mass index (BMI). Treatment effects were incorporated in the model as annual changes in eGFR and uACR per health state based on the findings of the EMPA-KIDNEY trial and were therefore only included for the health states for which data was available (i.e., populations included in the trial, so not for G1, G2A1, G3aA1, G5). Below we address the points made by the reviewer by population:*

- *eGFR ≥90 ml/min/1.73m2 (G1): As the reviewer correctly mentions, patients with an eGFR ≥90 ml/min/1.73m2 are not considered CKD patients and were also not included in the EMPA-Kidney trial. In our model, all G1 states were 0% at baseline and remained 0% throughout the time horizon as CKD is a progressive disease. Therefore, we made an adjustment to Figure 1 to clarify that G1 health state was not included in the model as this might understandably be perceived as confusing.*
- *eGFR <20 ml/min/1.73m2 (G5): These patients were not included in the EMPA-Kidney trial (SGLT2 inhibitor is also not recommended by KDIGO in this population), and therefore the proportion of patients in these health states was 0% at baseline. However, even though health state G5 was also not included in the model at baseline and no treatment effect was taken into account for this population, it is still possible for patients to transition to G5 health state, since CKD is a progressive disease.*
- *eGFR between 60-90 ml/min/1.73m2 without albuminuria or other signs of kidney damage (G2A1): As the reviewer mentions, these patients are not considered CKD patients according to the KDIGO guideline and were also not included in the EMPA-Kidney trial. However, as the baseline distribution was based on a combination of the proportion per eGFR category and uACR category, a proportion of patients of 7.7% G2 was multiplied by 20.1% in A1, resulting in a baseline population of 1.5% starting in health state G2A1 (eGFR between 60-90 ml/min/1.73m2 without albuminuria). The risk for CKD progression and complications for this group was – as for all health states – based on risk equation from literature. However, there is no treatment effect taken into account as treatment is not indicated for this group.*
- *Patients with an eGFR between 45-60 ml/min/1.73m2 without albuminuria (G3aA1): According to the KDIGO guideline SGLT2 inhibitors are recommended for patients with diabetes, CKD, and an eGFR* ≥*20 ml/min/1.73m2, patients with eGFR* ≥*20 with uACR* ≥*200 mg/g, or heart failure irrespective of albuminuria. Additionally, it is suggested for treating adults with eGFR 20-45 ml/min/1.73m2 without albuminuria. These recommendations match the inclusion criteria of the EMPA-Kidney trial, except for patients with an eGFR between 45-60 ml/min/1.73m2 with heart failure, irrespective of albuminuria. Therefore, there were no data as such available for this specific population and therefore, same as in the G5 and G2A1 groups, no treatment effect was taken into account.*

*Based on these points, we have made the following adjustments to the manuscript:*

- *We have now clarified that G1 was not considered in our model (Figure 1, lines 132-133):*
  - *“Health state G1 (eGFR > 90 ml/min/1.73m2) was not included in the model since this is not considered CKD according to KDIGO criteria.”*
  - *Figure 1:*

**Fig1. Health states, complications and events included in the CKD disease progression model.**

Abbreviations: A=KDIGO uACR category (in mg/g), AV=arteriovenous, BMD=bone and mineral disease, BMI=body mass index, BSI=blood stream infection, CKD=chronic kidney disease, CVD=cardiovascular disease, eGFR=estimated glomerular filtration rate, ESKD=end-stage kidney disease, G=KDIGO eGFR category (in mL/min/1.73^2^), HD=hemodialysis, HF=heart failure, HTN=hypertension, HS=health state, KDIGO=Kidney Disease: Improving Global Outcomes, MI=myocardial infarction, PAD=peripheral arterial disease, PD=peritoneal dialysis, RT=renal transplantation, TIA=transient ischemic attack, uACR=urine albumin-creatinine ratio.

*Even though this is not considered as CKD according to KDIGO criteria, an assumption has been made to include this in the model to model the disease progression, however, treatment effects were not considered in this group.

- *We now explain more clearly that the distribution over the health states as represented by the matrix in Figure 1 is calculated by multiplying the proportion of patients in each eGFR category by the proportion of patients in the uACR category (lines 133-135):*
  - *“The distribution over the different health states was calculated by multiplying the proportion of the patients in the EMPA-Kidney trial in each eGFR category (G2-G5) by the proportion in each uACR category (A1-A3).”*
- *We have now clarified that G5 is not considered at baseline, but is considered later in the model as the disease progresses (lines 160-164):*

*“At baseline, the distribution of eGFR and uACR levels were based on the levels of the included patients in the EMPA-Kidney trial (Table S1). The eGFR and uACR levels of included patients were used in a matrix to estimate the baseline distribution of health states. Due to the inclusion criteria of the EMPA-Kidney trial, no patients started in the G5 health states at baseline. However, it is possible for patients to transition to this health state in the model at a later stage.”*

- *We have now clarified in the legend under Figure 1 that G2A1 is included in our model to model the disease progression but there was no treatment effect included for this population (lines 150-151):*
  - *“*Even though this is not considered as CKD according to KDIGO criteria, an assumption has been made to include this in the model to model the disease progression, however, treatment effects were not considered in this group.”*
- *We have now clarified that the treatment effect was only included in the model for the populations included in the EMPA-Kidney trial (lines 166-167).*
  - *“No treatment effect was included for health states G2A1, G3aA1, and G5A1-3 as these populations were not included in the EMPA-Kidney trial.”*
- *We discuss in the limitations section that patients with eGFR between 45 and 60 ml/min/1.73m2 without were not included in the analysis, while these patients may, in accordance with KDIGO guidelines, be eligible for SGLT2i treatment in case they have heart failure as a comorbidity (lines 417-425):*
  - *“Besides, patients with eGFR 45-60 ml/min/m2 without albuminuria were not included in the EMPA-Kidney trial, despite having CKD according to KDIGO criteria. According to the KDIGO guideline SGLT2i are indicated in this population when they have heart failure as a comorbidity. Although this population was included in the model to properly model CKD progression, the treatment effect of empagliflozin was not included as there was no data from the EMPA-Kidney available for this group to support the effectiveness. Data on patients with eGFR between 45 and 60 ml/min, who meet KDIGO criteria but were excluded from the EMPA-kidney study, would provide valuable context and contribute to a more comprehensive understanding of empagliflozin's cost-effectiveness across different stages of CKD.”*

**Reviewer #2:**

**Slob et al present a detailed cost effectiveness analysis of empagliflozin in the Netherlands comparing standard of care for chronic kidney disease plus empagliflozin (empa) and standard of care alone (SoC). Using a Markov model they show that the cost effectiveness of SoC+empag versus SoC alone is a savings of 34,381 Euros. They show that SoC+empa adds an additional 1.22 life years compared to SoC alone. The authors present data on CKD in the Netherlands that shows prevalence around 12% but only 5.1% of the Dutch population is diagnosed. I have the following questions:**

- **Why was only empagliflozin studied and not the other major SGLT2 inhibitors (dapagliflozin and canagliflozin)? Are there cost analyses for them and how does the cost effectiveness of empagliflozin compare to the cost effectiveness of the others?**

*This is the first cost-effectiveness analysis of a SGLT2 inhibitor in patients with CKD in the Netherlands (lines 328-329): “To our knowledge, this is the first cost-effectiveness analysis of a SGLT2 inhibitor in patients with CKD in the Netherlands.” However, both empagliflozin and dapagliflozin are already reimbursed in the Netherlands, following a cost-effectiveness analysis included in the reimbursement dossier for dapagliflozin. That analysis showed very similar results as our analysis and concluded that dapagliflozin was dominant compared to SoC (lines 335-338): “Comparable to our results, a dominant ICER was reported by the National Health Care Institute (Zorginstituut Nederland) for dapagliflozin plus SoC versus SoC alone based on the DAPA-CKD trial for reimbursement purposes in the Netherlands”. Based on the reviewers comment we added the following sentence to the manuscript to emphasize that empagliflozin and dapagliflozin are comparable (lines 345-348):*

*“Given that both empagliflozin and dapagliflozin have similar effectiveness and pricing in the Netherlands, the differences in cost-effectiveness between them are expected to be minimal. However, small differences between the trials may explain part of the small discrepancies in cost-effectiveness results.”*

Additionally, in lines 355-357 we discuss the cost-effectiveness of canagliflozin: *“A cost-effectiveness study compared canagliflozin to dapagliflozin based on the DAPA-CKD and CREDENCE trials in patients with CKD and type 2 diabetes in Canada. They concluded that adding either canagliflozin or dapagliflozin to SoC was dominant over SoC alone.” However, canagliflozin is not reimbursed for CKD patients in the Netherlands, which we added to the manuscript to clarify (lines 357-358):*

*“However, canagliflozin is not reimbursed for CKD patients in the Netherlands.”*

*We added the following sentence to the discussion section to emphasize that all major SGLT2 inhibitors show similar results (lines 360-361):*

*“*Overall, these analyses demonstrate that all SGTL2 inhibitors in addition to SoC are likely dominant over SoC alone*.”*

- **What is the standard of care for CKD in the Netherland and does it vary from co-morbidity to co-morbidity (diabetes, heart failure, coronary artery disease. How does the analysis take account of the fact that standard cof care might differ with underlying co-morbidities. The authors provide the details from KDIGO (lines 82-93, but should include regimens specific to the Netherlands.**

*We thank the reviewer for this comment. We agree that the SoC might differ with underlying co-morbidities. Distinction between the SoC per co-morbidity might result in even more precise outcomes. We did consider the heterogeneity of the CKD population in modelling the disease progression. We used a microsimulation model which allows for individual patients to be randomly simulated and track individual disease histories over time. So, this does allow us to take into account the different underlying co-morbidities and their impact on patient outcomes, however we did not model the costs for each different regimen. We only considered the SoC costs for RAS inhibitors, to focus on CKD as the disease modelled rather than other comorbidities. However, we do agree it might be interesting to investigate the influence of other SoC costs on the ICER as well. Therefore, we added a scenario (scenario 8) in which we consider all the SoC costs for the comorbidities (as a weighted average) as reported in the EMPA-Kidney trial. This will still not fully represent the real-world Dutch setting, but this will result in a more accurate estimation of the costs with taking into account that SoC might differ per co-morbidity. The results of this scenario analysis show a slight increase in both the empagliflozin + SoC arm as well as in the SoC alone arm:*

- **How does Cost-effectiveness of empagliflozin and SoC differ across the European Union? What was the need to calculate cost effectiveness for empagliflozin specifically for the Netherlands?**

*There are not many cost-effectiveness studies about empagliflozin plus SoC in comparison to SoC alone in CKD. Very recently, a cost-effectiveness analysis was published in the UK, which also showed a dominant ICER for treatment with empagliflozin plus SoC compared to SoC alone: Ramos M, Gerlier L, Uster A, Muttram L, Frankel AH, Lamotte M. Cost-effectiveness of empagliflozin as add-on to standard of care for chronic kidney disease management in the United Kingdom. J Med Econ. 2024 Jan-Dec;27(1):777-785. doi: 10.1080/13696998.2024.2357041. Epub 2024 Jun 5. PMID: 38758099.*

*Furthermore, a cost-effectiveness study in Malaysia, Thailand and Vietnam also came to the same conclusion, with empagliflozin as add-on to SoC being cost-effective: Varghese, L., Chang, P. W., Juntarasiripas, S., Pham, H., & Uster, A. (2024). Cost-effectiveness of add-on empagliflozin versus standard of care in management of CKD in Malaysia, Thailand and Vietnam–findings from a modelling study assessing an EMPA-KIDNEY eligible population, using CKD progression model. Journal of Medical Economics, 27(1), 836-848*.

*Taking both studies into consideration, we added this information in the discussion section reading as follows (lines 329-333):*

*“Other recent cost-effectiveness analyses on empagliflozin have been conducted in the UK, Malaysia, Thailand and Vietnam which, like us, reported a dominant (UK, Malaysia, Thailand) or cost-effective (Vietnam) ICERs. Although input parameters were country specific, empagliflozin pricing was comparable to our study with £476,98 (€565,51) vs €546,55 for the UK while prices were somewhat lower in the Asian countries (ranging between €288.55 and €409.08 per year) [32,33].”*

*However there have been more analyses on dapagliflozin (which is very comparable to empagliflozin as discussed in a previous question) and canagliflozin. These cost-effectiveness analyses are discussed in lines 349-361 (see below).*

*“Another cost-effectiveness analysis based on the DAPA-CKD trial from the payer’s perspective was conducted for the UK, Germany and Spain showed ICERs of $8,280, $17,623 and $11,687 per QALY, with QALY gains of 0.82, 1.00 and 0.96 in the three countries respectively [35]. The difference compared with the results for the Dutch setting could include differences in modelling, input parameters, or pharmacoeconomic guidelines such as perspective and discounting. However, it would likely be a combination, as for example in our scenario from the payer’s perspective the ICER remained still dominant. A cost-effectiveness study compared canagliflozin to dapagliflozin based on the DAPA-CKD and CREDENCE trials in patients with CKD and type 2 diabetes in Canada [36]. They concluded that adding either canagliflozin or dapagliflozin to SoC was dominant over SoC alone. However, canagliflozin is not reimbursed for CKD patients in the Netherlands. The cost effectiveness of dapagliflozin has also been evaluated in non-diabetic CKD patients based on the non-diabetic cohort of the DAPA-CKD trial in US setting, showing an ICER of $60,000 per QALY [37]. Overall, these analyses demonstrate that all SGTL2 inhibitors in addition to SoC are likely dominant over SoC alone.”*

*We recognize that we did not address the need to do such analysis explicit enough. However, the KDIGO guideline explicitly mentions the need for economic evaluations, especially in people with CKD without diabetes and low levels of albuminuria to establish their level of cost-effectiveness. They also state: “From a healthcare system perspective, reducing cost burden of hospitalizations and dialysis is highly desirable, and QoL may be preserved longer from their avoidance. Specifics as to whether people bear the costs of these medications will be country-dependent.” Moreover, country-specific analyses are necessary as healthcare systems, guidelines, WTP-thresholds, and methods for calculating cost-effectiveness vary across countries. We therefore added the following sentences in the introduction section (lines 106-113):*

*“The KDIGO guideline highlights the need for economic evaluations, especially in people with CKD without diabetes and low levels of albuminuria to establish their level of cost-effectiveness. Reducing cost burden of hospitalizations and dialysis is highly desirable, and quality of life may be preserved longer from their avoidance. Specifics as to the costs of these medications offset the health gains will be country-dependent. Such country-specific analyses are necessary, as healthcare systems, guidelines, WTP-thresholds, and methods for calculating cost-effectiveness vary across countries. By focusing on the Netherlands, this study will highlight the value of empagliflozin within the Dutch healthcare context. The findings can help policymakers identify where its added value lies.”*
